# FOXO3 is a latent tumor suppressor for FOXO3-positive and cytoplasmic-type gastric cancer cells

**DOI:** 10.1038/s41388-021-01757-x

**Published:** 2021-04-01

**Authors:** Toshikatsu Tsuji, Yusuke Maeda, Kenji Kita, Kazuhiro Murakami, Hideyuki Saya, Hirofumi Takemura, Noriyuki Inaki, Masanobu Oshima, Hiroko Oshima

**Affiliations:** 1 Division of Genetics, Cancer Research Institute, Kanazawa University, Kanazawa, Japan; 2 Department of Thoracic, Cardiovascular and General Surgery, Kanazawa University, Kanazawa, Japan; 3 Department of Gastroenterological Surgery, Ishikawa Prefectural Central Hospital, Kanazawa, Japan; 4 Division of Gene Regulation, Institute for Advanced Medical Research (IAMR), Keio University School of Medicine, Tokyo, Japan; 5 Central Research Resource Branch, Cancer Research Institute, Kanazawa University, Kanazawa, Japan; 6 Division of Stem Cell Biology, Cancer Research Institute, Kanazawa University, Kanazawa, Japan; 7 Department of Gastroenterological Surgery, Kanazawa University, Kanazawa, Japan; 8 WPI Nano-Life Science Institute (Nano-LSI), Kanazawa University, Kanazawa, Japan

**Keywords:** Gastric cancer, Cell growth

## Abstract

FOXO3 is a member of the FOXO transcription factors thought to play a tumor-suppressor role in gastrointestinal cancer, while tumor-promoting function of FOXO3 has also been reported. These results suggest a context-dependent function of FOXO3 in tumor development. However, the relationship between the FOXO3 expression pattern and its role in tumorigenesis has not been elucidated. We examined the FOXO3 expression in 65 human primary gastric cancer and patient-derived xenograft tissues by immunohistochemistry and identified three subtypes according to subcellular localization: FOXO3-nuclear accumulated (FOXO3-Nuc), FOXO3-nuclear/cytoplasmic or cytoplasmic distributed (FOXO3-Cyt), and FOXO3-negative. In the FOXO3-Cyt gastric cancer cells, the expression of the constitutive active mutant FOXO3 (Act-ER FOXO3) induced the nuclear accumulation of FOXO3 and significantly suppressed colony formation and proliferation. The inhibition of the PI3K-AKT pathway by inhibitor treatment also suppressed the proliferation of FOXO3-Cyt gastric cancer cells, which was associated with the nuclear accumulation of endogenous FOXO3. Furthermore, the expression of Act-ER FOXO3 by an endogenous promoter significantly suppressed gastric tumorigenesis in *Gan* mice, a model of gastric cancer. Finally, treatment of FOXO3-Cyt human gastric cancer-derived organoids with an AKT inhibitor significantly suppressed the survival and proliferation. These results indicate that FOXO3 is a latent tumor suppressor for FOXO3-Cyt-type gastric cancer cells and that activation of the PI3K-AKT pathway protects this type of gastric cancer cell from FOXO3-mediated growth suppression via constitutive nuclear export. Thus, the inhibition of the PI3K-AKT pathway and nuclear translocation of endogenous FOXO3 may have therapeutic applications in the treatment of FOXO3-positive and cytoplasmic-type gastric cancer.

## Introduction

Gastric cancer is the fifth-most common type of cancer and the third leading cause of cancer-related death in the world [1, 2]. Despite recent advances in therapeutic strategies, the outcomes remain limited, particularly in advanced gastric cancer [3, 4]. While a genome-wide analysis identified frequently mutated genes in gastric cancers [5], the precise mechanism underlying the development and malignant progression of gastric cancer remains unclear. Besides genetic mutations, somatic copy number alterations in growth factor signaling, such as HER2, play an important role in gastric tumorigenesis [6]. Furthermore, *Helicobacter pylori* infection-induced inflammatory responses also activate growth factor signaling [7, 8]. Accordingly, an understanding of how such growth factor signaling regulates gastric tumorigenesis is important for the development of novel anti-gastric cancer drugs.

Among downstream growth factor signaling, the phosphatidyl inositol 3-kinase (PI3K)-AKT pathway is commonly hyperactivated in various cancers and is an important therapeutic target [9]. Activated AKT phosphorylates the forkhead box O (FOXO) family of transcription factors, resulting in the nuclear export of FOXOs [10]. Thus, the PI3K-AKT pathway negatively regulates FOXO transcriptional activity. Target molecules of FOXOs are involved in a wide range of biological functions, including stress responses, cell cycle inhibition, and apoptosis [11, 12]. FOXO3, a FOXO family member, induces the expression of pro-apoptotic protein BMF, resulting in apoptosis of epithelial cells. FOXO3 has therefore been considered as a tumor suppressor [13]. Consistently, simultaneous disruption of *Foxo1*, *Foxo3*, and *Foxo4* in mice resulted in the development of thymic lymphoma and hemangiomas, supporting a possible tumor-suppressor role of FOXOs [14]. Furthermore, *Foxo3* deficiency accelerated tumor development in a colitis-associated colon cancer mouse model [15].

However, other studies have reported that FOXO3 plays the opposite role in tumorigenesis. For example, FOXO3 promotes the malignant invasion of cancer cells by inducing the expression of matrix metalloproteinase [16] and is required for leukemia stem cell maintenance [17]. Furthermore, the simultaneous nuclear accumulation of FOXO3 and β-catenin promotes metastasis of colon cancer cells [18]. These results suggest that FOXO3 functions as a tumor promoter. Given such conflicting results regarding the role of FOXO proteins in tumorigenesis, it seems that FOXO3 is not merely a tumor suppressor—rather, it plays a complex role in tumorigenesis [19, 20]. Recently, it has been reported that FOXOs both suppress and support the progression of breast cancer cells, suggesting a role of FOXO3 in a cell context-dependent mechanism [21].

In the present study, we found three types of gastric cancers according to the expression and subcellular localization of FOXO3. Notably, gastric cancer cells with FOXO3-cytoplasmic distribution (FOXO3-Cyt) are sensitive to activated FOXO3-mediated growth suppression, and the inhibition of the PI3K-AKT pathway suppressed the survival and proliferation of gastric tumor cells associated with the nuclear accumulation of FOXO3. Furthermore, mouse genetic studies indicated that the nuclear accumulation of FOXO3 significantly suppressed gastric tumorigenesis. These results suggest that the nuclear translocation of endogenous FOXO3 through the inhibition of the PI3K-AKT pathway is a possible therapeutic strategy for FOXO3-Cyt-type gastric cancer.

## Results

### The distinct expression and distribution of FOXO3 in human gastric cancer tissues

In human gastrointestinal cancers, the FOXO3 expression is significantly downregulated, and this reduction is associated with an advanced stage [22, 23]. Furthermore, the high expression of FOXO3 in gastric cancer was correlated with a good prognosis, suggesting a tumor-suppressor role of FOXO3 [24]. However, the nuclear accumulation of FOXO3 and β-catenin promotes malignant progression of colon cancer [18], suggesting a cell-context dependent role of FOXO3 in tumorigenesis. We therefore examined the FOXO3 expression and subcellular localization in 50 human primary gastric cancer tissues by immunohistochemistry.

**Fig. 1:**
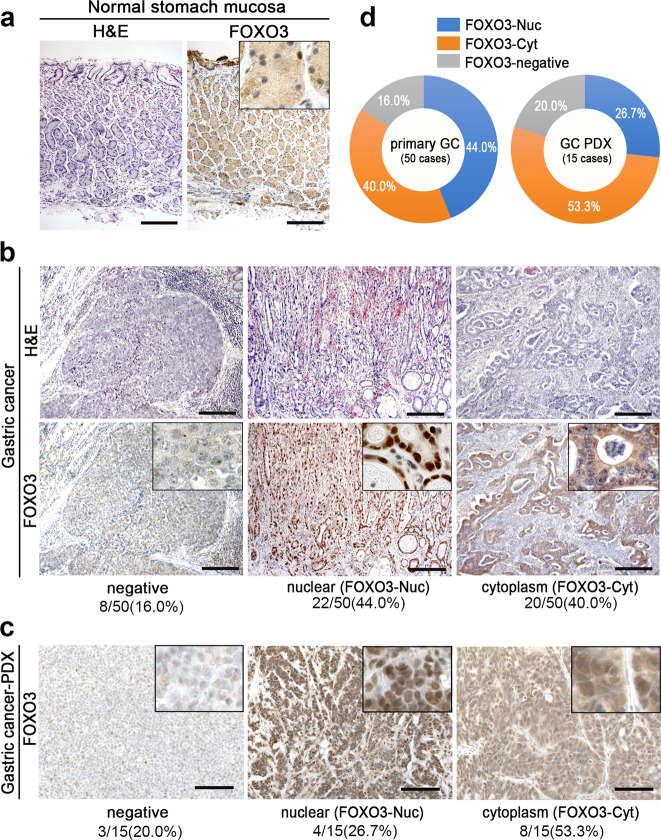
The expression and subcellular localization patterns of FOXO3 in human primary gastric cancer. **a** Representative photographs of histology sections (H&E) (*left*) and immunohistochemistry for FOXO3 (*right*) of non-tumor stomach tissue. The inset shows an enlarged image. Bars, 200 μm. **b** Representative photographs of histology sections (H&E) (*top*) and immunohistochemistry for FOXO3 (*bottom*) in human gastric cancer tissues: FOXO3-negative (*left*), FOXO3-positive and nuclear accumulated (FOXO3-Nuc type) (*center*), and FOXO3-positive and cytoplasmic distributed (FOXO3-Cyt type) (*right*). Insets show enlarged images. Bars, 200 μm. **c** Representative photographs of immunohistochemistry for FOXO3 in PDX tumors: FOXO3-negative (*left*), FOXO3-Nuc (*center*), and FOXO3-Cyt types (*right*). Insets show enlarged images. Bars, 100 μm. **d** The ratio of FOXO3-Nuc, FOXO3-Cyt, and FOXO3-negative human gastric cancer (*left*) and PDX tumors (*right)*. The images in (**a**) and (**b**) are representative of *n* = 50 independent human clinical samples. The images in (**c**) are representative of *n* = 15 independent PDX tumor samples.

In the non-tumor tissues of the stomach, weak FOXO3 staining was detected in the cytoplasm of epithelial cells (Fig. 1a). In 8 of 50 gastric cancer tissues (16.0%), FOXO3 expression was not detected (FOXO3-negative) (Fig. 1b, d). A database analyses indicated that deletion of the *FOXO3* gene was found in <1.6% of gastric cancers (Supplementary Fig. 1). On the other hand, it has been reported that the FOXO3 expression is regulated by microRNAs as well as post-translational modifications [25, 26]. It is thus possible that the FOXO3 expression is absent in FOXO3-negative type gastric cancers because of these mechanisms. In FOXO3-positive gastric cancers, a clear nuclear accumulation was found in 22 cases (44.0%) (FOXO3-Nuc), while FOXO3 was broadly distributed to the cytoplasm in the 20 remaining FOXO3-positive cases (40.0%) (FOXO3-Cyt). Although the ratio of FOXO3-positive gastric cancer is higher than that of FOXO3-negative in all cancer stages, we did not find a correlation between the FOXO3 subcellular localization and the cancer stages (Supplementary Table 1). We further performed immunohistochemical analyses of FOXO3 using 15 lines of gastric cancer PDX tumors. Consistent with the primary cancer, PDX tissues were classified into 3 types based on the FOXO3 expression patterns: FOXO3-negative (20.0%), FOXO3-Nuc (26.7%), and FOXO3-Cyt (53.3%) (Fig. 1c, d). In the present study, we examined whether or not FOXO3 plays a tumor-suppressor role in FOXO3-Cyt-type gastric cancer cells, in which FOXO3 is constitutively expressed and distributed to the cytoplasm.

### Suppression of FOXO3-Cyt-type cell proliferation by the nuclear accumulation of FOXO3

**Fig. 2:**
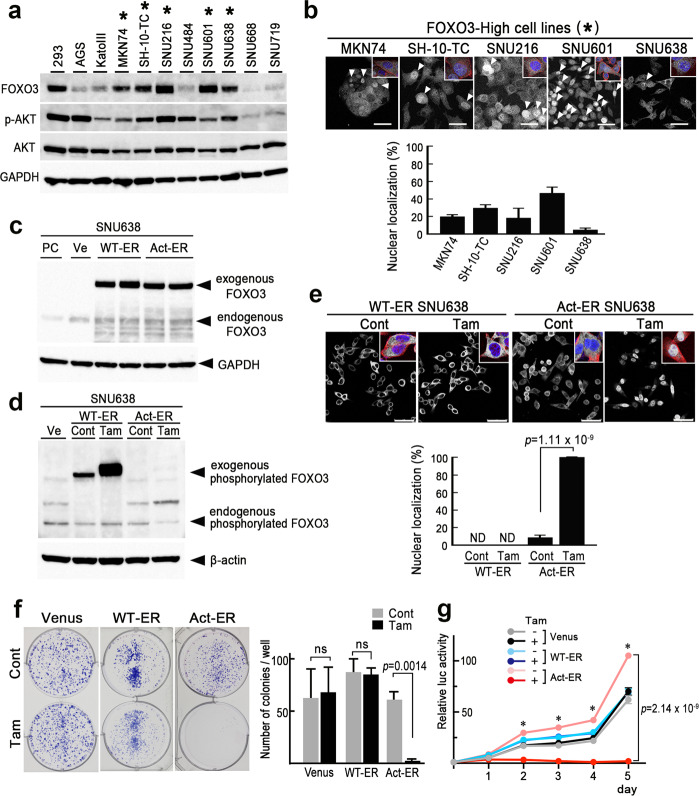
The growth suppression of FOXO3-Cyt-type gastric cancer cells by FOXO3 nuclear accumulation. **a** Immunoblotting of FOXO3, phosphorylated-AKT (p-AKT), and AKT in the indicated gastric cancer cell lines and 293 cells. GAPDH was used as the internal control. Asterisks indicate FOXO3-High gastric cancer cell lines. **b** Representative photographs of immunocytochemistry for FOXO3 in the indicated cell line (*top*). Insets show enlarged images of nuclear FOXO3-negative single FOXO3-High cells (asterisks in **a**) with Phalloidin (red) and DAPI (blue) staining. Arrowheads indicate cells with the nuclear accumulation of FOXO3. Bars, 50 μm. The images are representative of *n* = 3 independent cultures. The ratios of FOXO3-nuclear localized cells in the indicated gastric cancer cell lines are shown as a bar graph (mean ± s.d.) (*bottom)*. (*n* = 9 independent microscopic fields for each cell line). **c** Immunoblotting of exogenous FOXO3 (WT-ER and Act-ER) and endogenous FOXO3 in SNU638 cells. PC, Parental cells; Ve, Venus-expressing cells; WT-ER and Act-ER, wild-type FOXO3- and Act-ER FOXO3-expressing SNU638 cells, respectively. GAPDH was used as an internal control. **d** Immunoblotting of phosphorylated FOXO3 in control and Tam-treated WT-ER and Act-ER FOXO3-expressing SNU638 cells. β-Actin was used as an internal control. **e** Representative photographs of immunocytochemistry for FOXO3 in the tamoxifen-treated (Tam) and untreated (Cont) WT-ER and Act-ER FOXO3-expressing SNU638 cells (*top*). Insets show enlarged images of cells for FOXO3 staining with Phalloidin (red) and DAPI (blue) staining. Bars, 50 μm. The images are representative of *n* = 3 independent cultures. The ratios of FOXO3-nuclear localized cells in the control and Tam-treated WT-ER or Act-ER FOXO3-expressing SNU638 cells are shown as a bar graph (mean ± s.d.) (*bottom)*. (*n* = 9 independent microscopic fields for each cell line). A two-sided *t*-test was used to calculate statistical significance, and *p* value is provided. ND, not detected. **f** The results of a colony formation assay of Tam-treated (Tam) and untreated (Cont) SNU638 cells expressing Venus (*left*), WT-ER FOXO3 (*center*), and Act-ER FOXO3 (*right*) in 6-well plates are shown. The images are representative of *n* = 3 independent experiments. The mean colony numbers per well are shown in a bar graph (mean ± s.d.) (*right)*. A two-sided *t*-test was used to calculate statistical significance, and *p* value is provided. ns, not significant. **g** Relative cell proliferations examined by the luciferase (luc) activity in Tam-treated (+) or untreated (−) SNU638 cells expressing Venus, WT-ER FOXO3, and Act-ER FOXO3 are shown (*n* = 3 for each condition) (mean ± s.d.). The data at each day point were analyzed by one-way ANOVA test. Asterisks, *p* < 0.05. A two-sided *t*-test was used to calculate statistical difference between Tam(+) and Tam(-) Act-ER cells at day 5, and *p* value is *p*rovided. The immunoblotting experiments shown in (**a**), (**c**), and (**d**) were repeated three times with similar results.

We next examined the FOXO3 expression in gastric cancer cell lines by immunoblotting and found that 5 of the 10 lines showed an elevated FOXO3 expression (Fig. 2a, asterisks). The PI3K-AKT pathway has been shown to negatively regulate FOXO3 by phosphorylation, which results in the nuclear export of FOXO3 [10, 11]. Notably, phosphorylated AKT was detected in all FOXO3-High gastric cancer cell lines, suggesting the constitutive nuclear export of FOXO3 in these cells. We therefore examined the FOXO3 subcellular localization of these gastric cancer cells by immunocytochemistry (Fig. 2b). Notably, the majority of FOXO3-High cells showed nuclear/cytoplasmic or cytoplasmic distribution of FOXO3 (FOXO3-Cyt), whereas FOXO3 nuclear-accumulated (FOXO3-Nuc) cells were also found in a minor population (Fig. 2b*top, arrowheads*), and the mean ratios of FOXO3-Nuc cells in each line ranged from 5.3% (SNU638) to 46.6% (SNU601) (Fig. 2b*bottom*). These results prompted us to examine whether or not FOXO3-Cyt-type gastric cancer cells are sensitive to FOXO3-mediated growth inhibition.

To clarify this point, we constructed estrogen receptor (ER)-linked wild-type FOXO3 (WT-ER) and mutant FOXO3 (Act-ER)-expressing cells, using SNU638 and MKN74 cells. In these lines, tamoxifen (Tam) treatment induced the nuclear transportation of WT-ER FOXO3 and Act-ER FOXO3 by binding the ER at the C-terminal FOXO3. Furthermore, three AKT-phosphorylation sites were mutated in Act-ER FOXO3, which prevented the phosphorylation of FOXO3 by AKT [27]. The expression of exogenous FOXO3 proteins was confirmed by immunoblotting (Fig. 2c and Supplementary Fig. 2a). Tam treatment increased the level of phosphorylated WT-ER FOXO3 (Fig. 2d and Supplementary Fig. 2b), suggesting phosphorylation of nuclear transported WT-ER FOXO3. Moreover, FOXO3 was localized in the cytoplasm of both control and Tam-treated WT-ER FOXO3-expressing cells (Fig. 2e and Supplementary Fig. 2c). In contrast, in the Act-ER-expressing cells, Tam treatment induced the nuclear accumulation of FOXO3. These results indicate that AKT-induced phosphorylation is required for the constitutive nuclear export of FOXO3. Importantly, the cloning efficiency evaluated by colony formation in Act-ER-expressing cells was significantly suppressed by Tam treatment, while the WT-ER expression did not affect the colony formation (Fig. 2f and Supplementary Fig. 2d and e). Furthermore, the cell proliferation rate was significantly decreased in the Tam-treated Act-ER- but not WT-ER-expressing cells (Fig. 2g). Taken together, these results indicate that FOXO3-Cyt-type cells are sensitive to the activated FOXO3-induced inhibition of clonal expansion and proliferation.

### Suppression of FOXO3-Cyt-type cell proliferation by the inhibition of PI3K-AKT

**Fig. 3:**
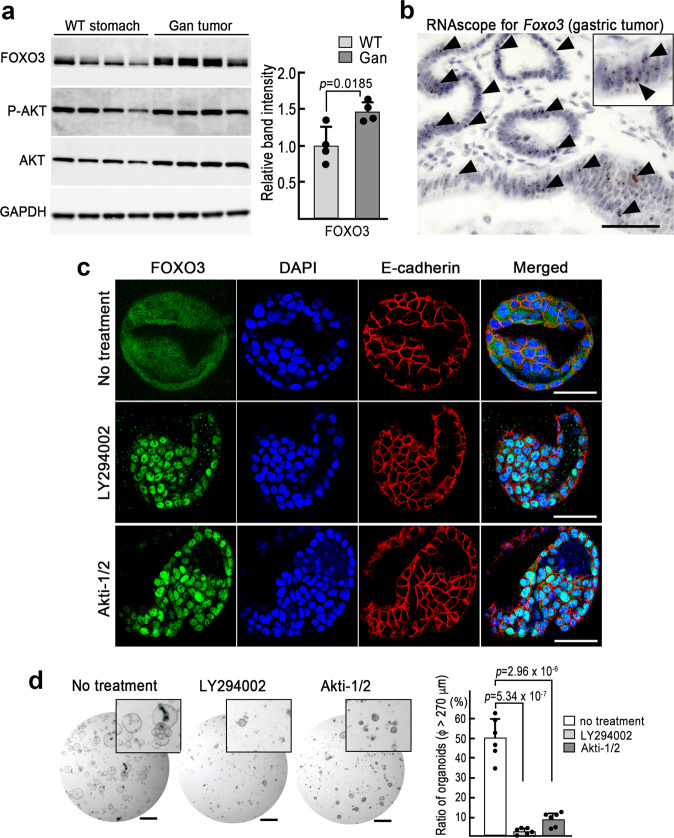
Growth suppression of mouse gastric tumor organoids by the inhibition of the PI3K-AKT signaling. **a** Immunoblotting of FOXO3, phosphorylated-AKT (p-AKT), and AKT in the wild-type mouse (WT) stomach (*n* = 4) and *Gan* mice gastric tumors (*n* = 4) (*left*). GAPDH was used as an internal control. The immunoblotting experiments were repeated three times with similar results. The mean relative band intensities of FOXO3 are shown in a bar graph (mean ± s.d.) (*right*). A two-sided *t*-test was used to calculate statistical significance, and *p* value is provided. **b** Representative photograph of in situ hybridization (RNAscope) to detect *Foxo3* mRNA in the gastric tumors of *Gan* mice. Arrowheads indicate positive signals. Inset shows an enlarged image. Bar, 20 μm. The image is a representative of *n* = 3 biologically independent animals. **c** Representative confocal microscopy images of immunocytochemistry for FOXO3 (green), E-cadherin (red), DAPI (blue), and merged images of *Gan* mouse tumor-derived organoids with no treatment control (*top*), treated with LY294002 (*middle*) and Akti-1/2 (*bottom*). Bars, 50 μm. The images are representative of *n* = 10 biologically independent organoids. **d** Representative dissection microscope images of *Gan* mouse tumor-derived organoids with no treatment control (*left*) and treated with LY294002 (*center*) and Akti-1/2 (*right*). Bars, 1 mm. Insets show enlarged images. The images are representative of *n* = 6 independent experiments. The ratios of organoids with φ > 270 μm are shown in a bar graph (mean ± s.d.). Individual data are shown with dots. A two-sided *t*-test was used to calculate statistical significance, and *p* values are provided.

We previously generated a gastric cancer mouse model (*Gan* mice) that shares histological characteristics and expression profiles with human intestinal-type gastric cancer [8, 28, 29]. In *Gan* mouse gastric tumors, the expression of FOXO3 was detected by immunoblotting, and the expression was significantly higher than that of wild-type mouse stomach (Fig. 3a). Phosphorylated AKT was also detected in both normal and tumor tissues. RNAscope in situ hybridization detected *Foxo3* mRNA in normal epithelial cells of the wild-type mouse stomach (Supplementary Fig. 3) and *Gan* mouse tumor cells (Fig. 3b). To further examine the role of FOXO3, we established organoids from *Gan* mouse gastric tumors. Immunocytochemistry confirmed that the expression of FOXO3 was distributed to the cytoplasm of organoid cells (Fig. 3c), indicating that *Gan* mice develop FOXO3-Cyt-type gastric tumors. Notably, treatment of the organoids with PI3K inhibitor (LY294002) or AKT inhibitor (Akti-1/2) resulted in the nuclear accumulation of FOXO3. Furthermore, organoid growth, as measured by organoid size, was significantly suppressed by treatment with either PI3K inhibitor or AKT inhibitor (Fig. 3d).

Moreover, treatment of FOXO3-Cyt-type gastric cancer cells (Fig. 2a, asterisks) with PI3K inhibitor or AKT inhibitor caused the accumulation of FOXO3 in nuclei, which was associated with the significant suppression of cell proliferation (Supplementary Fig. 4). Although the inhibition of the PI3K-AKT pathway suppresses various downstream signaling events aside from the suppression of the FOXO3 function, these results collectively suggest that the nuclear accumulation of FOXO3 is an important mechanism underlying PI3K-AKT inhibitor-induced growth suppression of FOXO3-Cyt-type gastric cancer cells.

### Resistance of FOXO3-Nuc-type cells to the inhibition of PI3K-AKT signaling

**Fig. 4:**
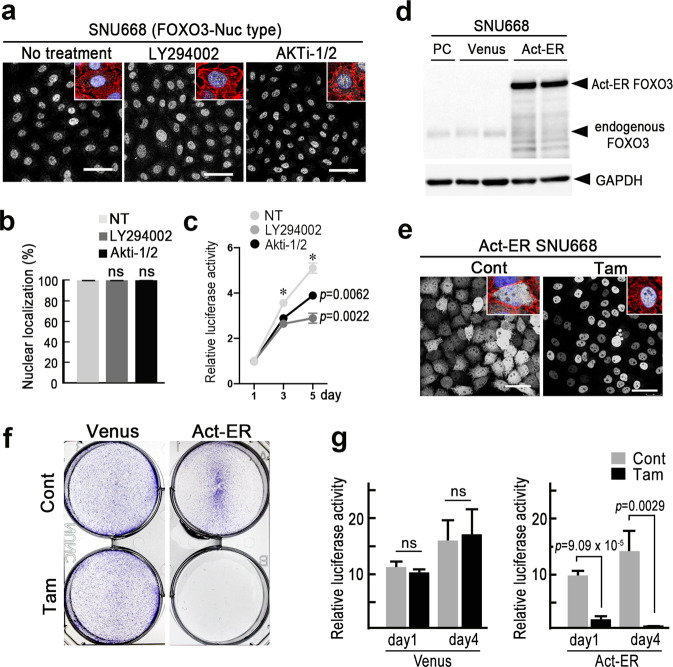
Resistance of FOXO3-Nuc-type gastric cancer cells to inhibition of PI3K-AKT signaling. **a** Representative photographs of immunocytochemistry for FOXO3 of the SNU668 cells treated with LY294002 (*center*) and AKTi-1/2 (*right*) and untreated control (*left*). Insets show enlarged images with Phalloidin (red) and DAPI (blue) staining. Bars, 50 μm. The images are representative of *n* = 3 independent experiments. **b** The ratios of FOXO3-nuclear localized cells in the SNU668 cells are shown as a bar graph (mean ± s.d.). (*n* = 9 independent microscopic fields for each treatment). A two-sided *t*-test was used to calculate statistical significance vs. NT. ns, not significant. **c** Relative cell proliferations examined by the luciferase activity in SNU668 cells with no treatment (NT) and treated with LY294002 or Akti-1/2 are shown (mean ± s.d.). The data at each day point were analyzed by one-way ANOVA test. A two-sided *t*-test was used to calculate statistical significance at day 5, and *p* values vs. NT are provided. **d** Immunoblotting of Act-ER FOXO3 and endogenous FOXO3 in SNU668 cells. PC, Parental cells; Venus, Venus-expressing cells; and Act-ER, Act-ER FOXO3-expressing cells. GAPDH was used as an internal control. **e** Representative photographs of immunocytochemistry for FOXO3 in the tamoxifen-treated (Tam) and untreated (Cont) Act-ER FOXO3-expressing SNU668 cells. Insets show enlarged images with Phalloidin (red) and DAPI (blue) staining. Bars, 50 μm. The images are representative of *n* = 3 independent cultures. **f** The results of a colony formation assay of Tam-treated (Tam) and untreated (Cont) SNU668 cells expressing Venus (*left*) and Act-ER FOXO3 (*right*) in 6-well plates are shown. The images are representative of *n* = 3 independent experiments. **g** Relative cell proliferations examined by luciferase activity at day 1 and day 4 in Tam-treated (Tam) or untreated (Cont) SNU668 cells expressing Venus (*left*) and Act-ER FOXO3 (*right*) are shown (mean ± s.d.). A two-sided *t*-test was used to calculate statistical significance, and *p* values are provided. ns not significant.

We found that one of the FOXO3-Low cell line SNU668 showed nuclear FOXO3 localization with 100% efficiency (Figs. 2a and 4a). We thus examined whether or not FOXO3 can function as a tumor suppressor in FOXO3-Nuc-type gastric cancer cells. We confirmed that FOXO3 was nuclear-localized in SNU668 cells at 100% incidence when treated with a PI3K or AKT inhibitor, just as in no-inhibitor-treated controls (Fig. 4a, b). In contrast to FOXO3-Cyt-type cells, SNU668 cells survived and proliferated in the presence of either a PI3K or AKT inhibitor, although the proliferation rate was significantly decreased (Fig. 4c). We next established exogenous Act-ER FOXO3-expressing SNU668 cells (Fig. 4d). In this cell line, Tam treatment induced clear nuclear accumulation of exogenous FOXO3 (Fig. 4e). Notably, Tam-treated Act-ER-expressing SNU668 cells showed significant suppression of colony formation and proliferation (Fig. 4f, g). These results suggest that FOXO3-Nuc-type gastric cancer cells can survive and proliferate with endogenous level of FOXO3, so the maintenance of a low FOXO3 expression is important for escaping from the tumor suppressor role in FOXO3-Nuc-type cells.

### Construction of active mutant FOXO3-expressing mouse model

**Fig. 5:**
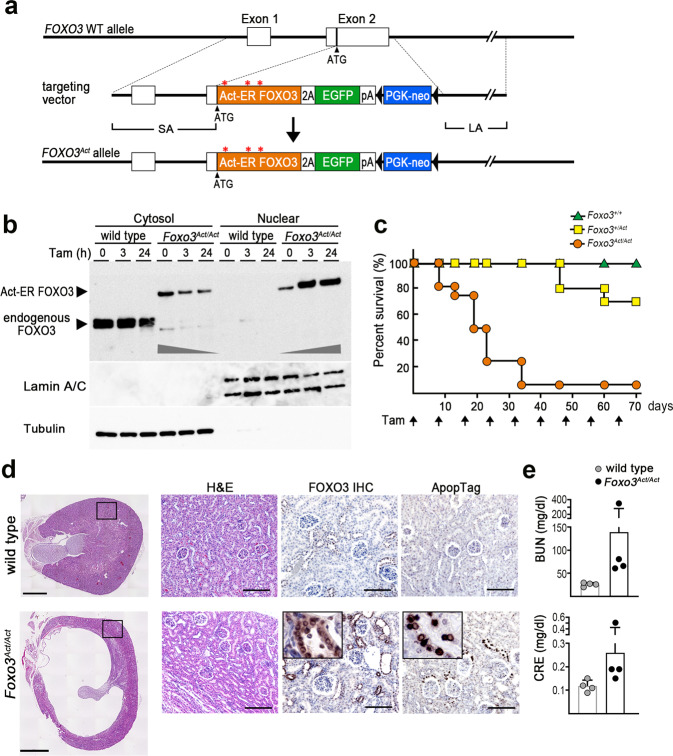
Construction of Act-ER FOXO3-expressing mice. **a** Schematic drawing of the strategy for generating Act-ER FOXO3-expressing mice by homologous recombination. The cDNA of Act-ER FOXO3 linked with 2A peptide and EGFP cDNA was introduced into exon 2 of endogenous *Foxo3* by homologous recombination. The short arm (SA) and long arm (LA) of the targeting vector. Black triangles indicate loxP. Asterisks indicate mutations at AKT recognition sites. **b** Immunoblotting of Act-ER FOXO3 and endogenous FOXO3 in cytosol and nuclear fractions of wild-type and *Foxo3*^*Act/Act*^ mouse gastric epithelial cells treated with tamoxifen (Tam) are shown. Lamin A/C and tubulin were used as internal controls of nuclear and cytosol proteins, respectively. The immunoblotting experiments were repeated three times with similar results. **c** The percent survival of *Foxo3*^*+/+*^*, Foxo3*^*+/Act*^, and *Foxo3*^*Act/Act*^ mice after treatment with Tam. Arrows indicate the timing of Tam treatment. (*n* = 10, 10, and 16 for *Foxo3*^*+/+*^*, Foxo3*^*+/Act*^, and *Foxo3*^*Act/Act*^ mice, respectively). **d** Representative photographs of kidney histology sections (H&E) (low-powered magnification) (*left*) and enlarged images of the boxed area (H&E) and serial sections for FOXO3 immunohistochemistry (IHC) and Apop Tag staining (*right*) are shown. Wild-type (*top*) and Tam-treated *Foxo3*^*Act/Act*^ mice (*bottom*). Insets indicate enlarged images. Bars, 1 mm (*left*) and 100 μm (*right*). The images are representative of *n* = 4 biologically independent animals for each genotype. **e** The blood urea nitrogen (BUN) levels (*top*) and serum creatinine (CRE) levels (*bottom*) of wild-type and *Foxo3*^*Act/Act*^ mice are shown in bar graphs (mean ± s.d.). Individual data are shown with dots.

To further examine the role of FOXO3 in gastric tumorigenesis, we constructed a conditional FOXO3 nuclear accumulation mouse model (*Foxo3*^*Act*^ mice) by homologous recombination in ES cells, in which mutant Act-ER FOXO3 is expressed under the control of an endogenous *Foxo3* promoter (Fig. 5a). To confirm the Tam-induced FOXO3 nuclear accumulation, primary cultured gastric epithelial cells from wild-type and *Foxo3*^*Act/Act*^ mice were examined by immunoblotting. In the wild-type mouse stomach cells, endogenous FOXO3 was predominantly detected in cytoplasm (Fig. 5b). In contrast, in the *Foxo3*^*Ac/Act*^ mouse-derived gastric epithelial cells, the level of Act-ER FOXO3 in the cytoplasm was decreased by Tam treatment, while nuclear Act-ER FOXO3 was increased, indicating the Tam-induced nuclear accumulation of Act-ER FOXO3 in gastric epithelial cells.

When *Foxo3*^*Act/Act*^, *Foxo3*^*+/Act*^, and *Foxo3*^*+/+*^ mice were treated with Tam, *Foxo3*^*Act/Act*^ mice showed a moribund phenotype from 8 days after treatment, and >80% of mice were euthanized by 35 days because of a severe phenotype (Fig. 5c). Notably, Tam-treated homozygous *Foxo3*^*Act/Act*^ mice showed severe kidney tubular atrophy (Fig. 5d, *left*). The nuclear accumulation of FOXO3 was detected in tubular epithelial cells, which was associated with apoptosis (Fig. 5d, *right*). Furthermore, the levels of blood urea nitrogen (BUN) and serum creatinine (CRE) in Tam-treated *Foxo3*^*Act/Act*^ mice were drastically increased compared to wild-type mice, indicating an impaired kidney function (Fig. 5e). Although, hypoxia-induced FOXO3 activation leads to tubular regeneration in acute kidney injury [30], the constitutive activation of FOXO3 in kidney tubular cells may cause growth arrest and apoptosis. In contrast, approximately 70% of Tam-treated *Foxo3*^*+/Act*^ mice survived for more than 10 weeks. Accordingly, we used *Foxo3*^*+/Act*^ mice for our next crossing experiments.

### Suppression of gastric tumorigenesis by FOXO3 nuclear accumulation in Gan mice

**Fig. 6:**
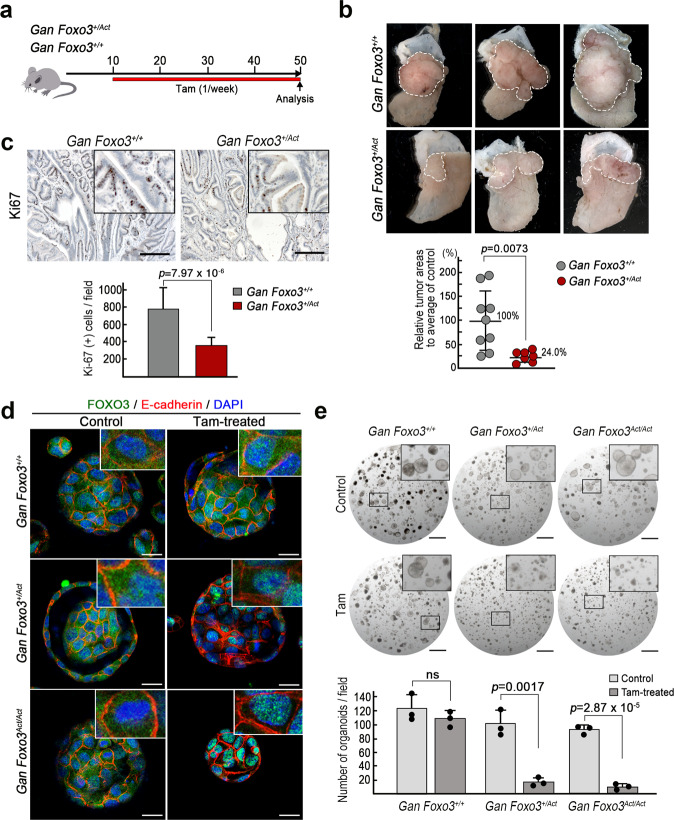
The suppression of *Gan* mouse gastric tumorigenesis by the nuclear accumulation of FOXO3. **a** A schematic illustration of the Tam treatment strategy for *Gan Foxo3*^*+/Act*^ and *Gan Foxo3*^*+/+*^ mice. The red bar indicates the Tam treatment period. **b** Representative macroscopic photographs of *Gan Foxo3*^*+/Act*^ and *Gan Foxo3*^*+/+*^ mouse gastric tumors at 50 weeks of age (*top*). Dashed lines indicate tumor areas. The images are representative of *n* = 9 and *n* = 7 biologically independent *Gan Foxo3*^*+/+*^ and *Gan Foxo3*^*+/Act*^ mice, respectively. The tumor area in each genotype mouse relative to the mean level of *Gan Foxo3*^*+/+*^ mouse tumors is shown in a dot graph (mean ± s.d.) (*bottom*). A two-sided *t*-test was used to calculate statistical significance, and *p* value is provided. **c** Representative photographs of immunohistochemistry for Ki67 of *Gan FOXO3*^*+/+*^ (*top left*) and *Gan Foxo3*^*+/Act*^ mouse tumors (*top right*). Bars, 200 μm. Insets show enlarged images. The images are representative of *n* = 4 biologically independent animals for each genotype. The mean numbers of Ki67-positive cells per microscopic fields (*n* = 12 fields for each genotype) are indicated (mean ± s.d.) (*bottom*). A two-sided *t*-test was used to calculate statistical significance, and *p* value is provided. **d** Representative merged images of immunocytochemistry for FOXO3 (green), E-cadherin (red), and DAPI (blue) of tumor-derived organoids from *Gan Foxo3*^*+/+*^ (*top*), *Gan Foxo3*^*+/Act*^ (*middle*), and *Gan Foxo3*^*Act/Act*^ mice (*bottom*). Organoids were treated with Tam after seeded in Matrigel. Bars, 25 μm. Insets show enlarged images. The images are representative of *n* = 10 biologically independent organoids. **e** Representative dissection microscope images of the tumor-derived organoids from *Gan Foxo3*^*+/+*^ (*left*), *Gan Foxo3*^*+/Act*^ (*center*), and *Gan Foxo3*^*Act/Act*^ mice (*right*) without Tam treatment (Control) (*top*) and with Tam treatment (*bottom*). Bars, 1 mm. Insets show enlarged images. The images are representative of *n* = 3 independent experiments. The mean numbers of organoids per microscopic field are indicated in a bar graph (mean ± s.d.) (*bottom*). Individual data are shown with dots. A two-sided *t*-test was used to calculate statistical significance, and *p* values are provided. ns not significant.

We generated *Gan Foxo3*^*+/Act*^ compound mice by crossing, and mice were treated with Tam for 40 weeks from 10 weeks of age (Fig. 6a). Importantly, gastric tumor development was suppressed in *Gan Foxo3*^*+/Act*^ mice, and the mean tumor area measured on the histology sections decreased significantly to 24.0% of that in control *Gan Foxo3*^*+/+*^ mice (Fig. 6b and Supplementary Fig. 5). Consistently, the mean Ki67 labeling index in *Gan Foxo3*^*+/Act*^ mouse tumors was significantly decreased compared with that in *Gan Foxo3*^*+/+*^ mice (Fig. 6c). These results indicate that the nuclear accumulation of a physiological level of FOXO3 transcribed by an endogenous promoter suppresses FOXO3-Cyt-type gastric tumor development.

To further examine the tumor-suppressor role of FOXO3, we established organoids from gastric tumors of Tam-untreated *Gan Foxo3*^*+/+*^, *Gan Foxo3*^*+/Act*^*,* and *Gan Foxo3*^*Act/Act*^ mice. Immunocytochemical analyses showed that the FOXO3 was distributed to the cytoplasm in all *Foxo3* genotype organoid cells before Tam treatment (Fig. 6d left). In contrast, Tam treatment induced the FOXO3 nuclear accumulation in both *Gan Foxo3*^*+/Act*^ and *Gan Foxo3*^*Act/Act*^ tumor organoids, which was not found in Tam-treated *Gan Foxo3*^*+/+*^ organoids (Fig. 6d right). Consistently, the expression of FOXO3-target genes *Cdkn1b* and *Bcl2l11* was significantly increased in the Tam-treated *Gan Foxo3*^*Act/Act*^ and *Gan Foxo3*^*+/Act*^ organoids, with the exception of *Cdkn1b* in *Gan Foxo3*^*+/Act*^ organoids, suggesting that the FOXO3 transcription activity in *Gan Foxo3*^*Act/Act*^ cells is higher than that in *Gan Foxo3*^*+/Act*^ (Supplementary Fig. 6a). Furthermore, Tam treatment significantly suppressed the organoid development of *Gan Foxo3*^*+/Act*^ and *Gan Foxo3*^*Act/Act*^ tumor cells when organoids were enzymatically dissociated to single cells and passaged (Fig. 6e). Importantly, when organoids were mechanically dissociated by pipetting to cell clumps, the mean numbers of *Gan Foxo3*^*+/Act*^ and *Gan Foxo3*^*Act/Act*^ organoids were slightly decreased by Tam treatment, although not to a significant degree (Supplementary Fig. 6b). These results suggest that the nuclear accumulation of FOXO3 effectively suppresses the organoid formation when tumor cells lose cell-cell contact. In contrast, when tumor cells form a glandular structure, cell-cell adhesion may contribute to resistance to FOXO3-induced growth suppression.

### Suppression of gastric tumorigenesis by inhibition of PI3K-AKT pathway

**Fig. 7:**
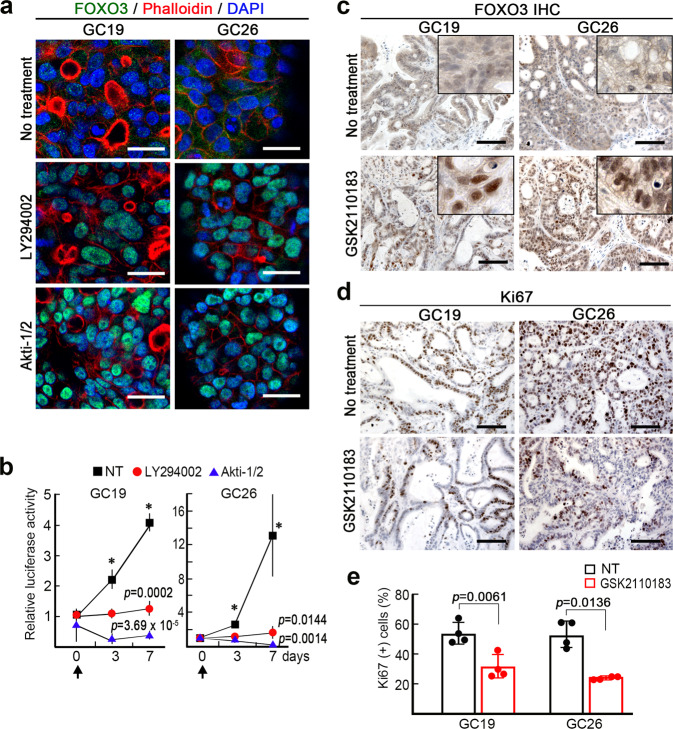
Suppression of tumorigenesis of human gastric cancer cells by the inhibition of PI3K-AKT. **a** Representative merged images of immunocytochemistry for FOXO3 (green), Phalloidin (red), and DAPI (blue) of GC19 (*left*) and GC26 (*right*) organoids are shown. No treatment (*top*) and treated with LY294002 (*middle*) and Akti-1/2 (*bottom*). Bars, 25 μm. The images are representative of *n* = 10 biologically independent organoids. **b** The findings of cell proliferation assays of GC19 (*left*) and GC26 (*right*) with no treatment (NT) and treated with LY294002 and Akti-1/2 are shown as line graphs (mean ± s.d.). Cells were continuously treated with LY294002 and Akti-1/2 from day 0 (arrows). The data at each day point were analyzed by one-way ANOVA test. Asterisks, *p* < 0.05. A two-sided *t*-test was used to calculate statistical significance at day 7, and *p* values vs. NT are provided. **c** Representative photographs of immunohistochemistry for FOXO3 of the GC19 (*left*) and GC26 (*right*) PDX tumors with no treatment (*top*) and treated with GSK2110183 (*bottom*) are shown. Bars, 100 μm. Insets show enlarged images. **d** Representative photographs of immunohistochemistry for Ki67 in GC19 (*left*) and GC26 (*right*) PDX tumors with no treatment (*top*) and treated with GSK2110183 (*bottom*) are shown. Bars, 100 μm. The images in ***c*** and ***d*** are representative of *n* = 4 biologically independent animals. **e** The ratios of Ki67-positive cells in PDX tumors with no treatment (NT) and treated with GSK2110183 are shown as a bar graph (mean ± s.d.). Individual data are shown with dots. A two-sided *t*-test was used to calculate statistical significance, and *p* values are provided.

We finally examined the effect of the inhibition of PI3K-AKT signaling in FOXO3-Cyt-type human gastric cancer using the gastric cancer-derived organoid lines GC19 and GC26. Immunocytochemistry showed that FOXO3 was weakly expressed in the cytoplasm of these organoid cells; however, the treatment of the organoids with PI3K inhibitor or AKT inhibitor induced the clear nuclear accumulation of FOXO3 in both organoid cells (Fig. 7a). As expected, treatment of organoids with a PI3K or AKT inhibitor significantly suppressed the survival and proliferation of enzymatically dissociated organoid cells (Fig. 7b). Finally, we treated PDX models constructed with GC19 and GC26 tumor tissues with the AKT inhibitor Afuresertib (GSK2110183). Notably, Afuresertib treatment induced the clear nuclear accumulation of FOXO3 in the PDX tumor cells (Fig. 7c), and the Ki67 labeling indices of tumor cells decreased significantly (Fig. 7d, e). These results confirmed that FOXO3-Cyt-type gastric cancer cells are sensitive to growth suppression mediated by the nuclear accumulation of endogenous FOXO3. It is therefore possible that treatment of FOXO3-Cyt-type gastric cancer with a PI3K-AKT inhibitor is effective for suppressing the survival or proliferation through the nuclear translocation of FOXO3.

## Discussion

In the present study, we found three types of gastric cancers according to the expression and subcellular localization of FOXO3: the FOXO3-Nuc, FOXO3-Cyt, and FOXO3-negative types. Among the three subtypes of gastric cancer, we show here that FOXO3-Nuc-type cells are resistant to endogenous FOXO3-induced growth suppression. However, the forced expression of Act-ER FOXO3 suppressed the survival and proliferation possibly by increased levels of active FOXO3. These results suggest that keeping the FOXO3 expression at low level is important for allowing it to escape from tumor suppressor role in FOXO3-Nuc-type cells. In contrast, in FOXO3-Cyt gastric cancer cells, we showed that the nuclear translocation of FOXO3 by the expression of Act-ER FOXO3 from an endogenous promoter or inhibition of PI3K-AKT signaling suppressed the colony formation and proliferation of gastric tumor cells. Therefore, endogenous FOXO3 can play a tumor-suppressor role via its nuclear accumulation in FOXO3-Cyt-type gastric cancer. We did not detect any correlation between the FOXO3 localization and the malignancy stage; the FOXO3 expression and its subcellular localization therefore cannot be used as a prognostic marker for gastric cancer. However, such information can help clarify the sensitivity of gastric cancer cells to FOXO3-mediated growth suppression, which may aid in establishing a therapeutic strategy using PI3K-AKT inhibitors or other compounds that induce the nuclear accumulation or activation of FOXO3.

In this study, we used a constitutive active FOXO3 model to examine the tumor suppressor function of FOXO3. However, it has been shown that FOXO3 activity is regulated not only by nuclear import and export but also by binding 14-3-3 protein, resulting in the regulation of DNA binding and protein stabilization [31]. Accordingly, it will be important to confirm the tumor suppressor role of FOXO3 by a different approach, such as loss of FOXO3 function model. Furthermore, we were unable to examine the role of FOXO3 in the malignant progression of gastric cancer, as *Gan* mice do not develop metastasis. Therefore, to examine whether FOXO3 acts as a tumor suppressor or promoter in metastasis, the generation of a malignant model will be important.

In the present study, a majority of cells in the FOXO3-high gastric cancer cell lines were FOXO3-Cyt cells, with FOXO3-Nuc cells included as a minor population, suggesting that shuttling FOXO3 between the cytoplasm and nucleus, and nuclear-localized FOXO3 in a minor population plays other important roles. Reactive oxygen species (ROS) signaling induces the nuclear accumulation of FOXO proteins [32], and FOXO3 plays a protective role against ROS stress [33, 34]. We previously showed that the CD44 variant form is required for gastric tumorigenesis through the protection of tumor cells from ROS-induced damage [35]. In contrast, the basal level of ROS signaling is important for maintaining gastric tumor cells in an undifferentiated state [36]. Thus, the fine-tuning of the response to ROS signaling is important for the cancer cell survival and proliferation [37]. It is therefore possible that, at low levels, FOXO3 protects cells from ROS stress by nuclear translocation, while increased nuclear FOXO3 causes growth suppression in these cells.

Intriguingly, the FOXO3 nuclear accumulation suppressed the survival and proliferation of organoid cells when organoids were enzymatically dissociated into single cells. However, when the same organoids were mechanically dissociated into cell clumps, the effect of FOXO3 on growth suppression was limited. The disruption of cell-cell or cell-extra cellular matrix (ECM) contact has been shown to cause epithelial cell death, a process known as anoikis [38]. Furthermore, the inhibition of PI3K in breast and ovarian cancer-derived spheroids only results in apoptosis inside of spheroids, with the ECM-attached outer cells being resistant to anoikis [39]. These results suggest that FOXO3 activation is not sufficient for the induction of apoptosis if cancer cells maintain cell-cell or cell-ECM contact. Accordingly, with the FOXO3 nuclear accumulation strategy against cancer cells, it may be efficient to target solitary and dormant cells that are detached from the primary cancer and disseminated to distant organs.

In conclusion, we classified gastric cancer into three types based on the FOXO3 expression pattern. We found that the nuclear accumulation of FOXO3 causes growth suppression in FOXO3-Cyt-type gastric cancer, indicating a tumor-suppressor role of FOXO3 in this type of gastric cancer. Furthermore, the inhibition of the PI3K-AKT pathway led to the suppression of the survival and proliferation of FOXO3-Cyt-type gastric cancer cells, which is associated with the clear nuclear accumulation of FOXO3. Accordingly, the present results suggest that the inhibition of PI3K-AKT signaling and nuclear transportation of the endogenous FOXO3 are potential therapeutic strategies for FOXO3-Cyt-type gastric cancer.

## Materials and methods

### Patients and gastric cancer specimens

Human primary gastric cancer samples were obtained from 65 patients who underwent surgical resection at Ishikawa Prefectural Central Hospital, Japan. Fifty specimens were used for immunohistochemistry, and 15 were used to generate the patient-derived xenograft (PDX) model.

All experiments using human samples were approved by the Human Genome/Gene Analysis Research Ethics Committee of Kanazawa University (2016-086-433), and written informed consent was obtained from the patients.

### Gastric cancer PDX model

Gastric cancer specimens were cut into small pieces of approximately 3–5 mm and subcutaneously transplanted into immunodeficient Crlj:SHO-*Prkdc*^*scid*^*Hr*^*hr*^ mice (SHO mice; Charles River, Yokohama, Japan). When the tumor size reached 1.0–1.5 cm in diameter, tumors were collected, cut into small pieces, and subcutaneously transplanted into new SHO mice.

### Histology and immunohistochemistry

Tissues were fixed in 4% paraformaldehyde, embedded in paraffin, and sectioned at 4*-*µm thickness. The sections were stained with H&E or processed for immunohistochemistry. The height of the mouse gastric tumors was measured using H&E sections and the relative tumor height was calculated. Antibodies used for immunohistochemistry are indicated in Supplementary Information. Immunohistochemistry staining signals were visualized using an ImmPACT DAB Peroxidase Substrate Kit (Vector Laboratories, Burlingame, CA). The numbers of Ki67-positive cells and total epithelial cells were counted in three microscopic fields for each mouse (*n* = 4), and the mean Ki67 labeling indices were calculated. Apoptosis was detected using an ApopTag peroxidase in situ apoptosis detection kit (Chemicon, Temecula, CA).

### Cell culture experiments

The gastric cancer cell lines MKN74, Kato-III, SH-10-TC (Riken Bioresource Center, Tsukuba, Japan), AGS (ATCC), SNU216, SNU484, SNU601, SNU638, SNU668, and SNU719 (Korean Cell Line Bank, Seoul, Korea) were cultured in RPMI1640 or DMEM supplemented with 10% FBS. All cell lines were authenticated by an isoenzyme analysis or short tandem repeat analysis and initially expanded and cryopreserved within one month of receipt. Cells were used within three months after thawing frozen vials. The construction of WT-ER FOXO3- and Act-ER FOXO3-expressing cells is indicated in Supplementary Information.

For the colony formation assay, 2 × 10^3^ cells were cultured in a 6-well plate in the presence or absence of tamoxifen (Sigma-Aldrich) at 1 μM for 10 days. Cells were then stained with Giemsa solution, and the colony numbers per well were counted. For the cell proliferation analysis, 1 × 10^3^ cells were seeded in a 96-well plate, and the cell viability was examined with a Cell Titer-Glo Cell Viability Assay (Promega, Madison, WI). The luciferase activity was measured by a Centros XS^3^ LB960 (Berthold Technologies, Bad Wildbad, Germany). All cell culture experiments were repeated three times. Mycoplasma testing was performed using a direct immunofluorescence test.

### Organoid culture experiments

Organoids were prepared from mouse gastric tumors according to the published method [40]. The organoid culture medium is described in Supplementary Information. In brief, dissected tumor tissues were soaked in 5 mM EDTA at 4 °C for 2 h, and the isolated glands were embedded in growth-factor-reduced (GFR) Matrigel (Corning, Corning, NY) and cultured supplemented with 50% of L-WRN-conditioned medium [41]. For enzymatic passaging, organoids were treated with trypsin, and dissociated cells were seeded in Matrigel. For mechanical passaging, collected organoids were roughly dissociated by pipetting and transferred to Matrigel. The number of organoids was counted (*n* = 6 and *n* = 3 for *Gan* mouse organoids and *Gan Foxo3* compound mutant mouse organoids, respectively), and the mean ratios were calculated. To inhibit PI3K and AKT, organoid cells were seeded in Matrigel and cultured for three days and then treated with LY294002 and Akti-1/2 (Abcam) for two days.

To establish human gastric cancer-derived organoids, minced tumor tissues were washed in PBS with 2.5 μg/mL amphotericin (FUJIFILM Wako, Osaka, Japan), filtered with a 100-μm-pore filter, embedded in GFR Matrigel (Corning), and cultured in the same medium as mouse organoids.

### Reagents

Cells and organoids were cultured with LY294002 (Abcam) at 25 μM to inhibit PI3K and with Akti-1/2 (Abcam) at 10 μM to inhibit AKT. To assess the nuclear accumulation of WT-ER FOXO3 and Act-ER FOXO3, cells and organoids were treated with 1 μM 4-hydroxytamoxifen (Sigma-Aldrich). To inhibit AKT in vivo, Afuresertib (GSK2110183; 100 mg/kg/day, per oral) was administered for 5 continuous days/week from 28 to 63 days (5 weeks) after transplantation.

### Immunoblotting analyses

Cells and tissues were homogenized in lysis buffer, and 10 μg of the protein samples was separated using a 7.5 or 10% SDS-polyacrylamide gel. The antibodies used for immunoblotting are indicated in Supplementary Information. The ECL detection system (GE Healthcare, Buckinghamshire, UK) was used to detect the immunoblotting signals. The cytosol and nuclear protein samples were separately prepared from the primary cultures of mouse gastric epithelial cells for immunoblotting. The relative band intensities were measured using the ImageJ software program (NIH, National Institutes of Health).

### Immunocytochemistry

Gastric cancer cells and organoids were fixed in 4% PBS-formaldehyde and permeabilized with 0.1% Triton X-100. Antibodies used for immunocytochemistry are indicated in Supplementary Information. DAPI was used for counter staining. Fluorescent immunostaining images of organoids were obtained using a TCS SP8 confocal microscope (Leica Microsystems, Wetzlar, Germany). The numbers of FOXO3-nuclear-localized cells and total cells were counted in three independent immunocytochemistry microscopic fields for each culture experiment, and the mean ratios of the FOXO3 nuclear accumulation were calculated.

### *RNAscope* in situ *hybridization*

*Foxo3* mRNA was detected by in situ hybridization using an RNAscope 2.5 HD Assay-BROWN (Advanced Cell Diagnostics, Hayward, CA). Tissues were fixed in 4% PFA overnight at 4 °C and embedded in paraffin blocks. Sections were retrieved in boiling buffer and treated with protease for 30 min, and in situ hybridization was performed on 6-µm-thick sections according to the manufacturer’s instructions.

### Reverse transcription (RT)-polymerase chain reaction (PCR)

To examine the mRNA levels, organoids were treated with Tam for five days, and total RNA was extracted using an RNeasy Plus Micro Kit (Qiagen, Hilden, Germany), reverse-transcribed using a PrimeScript RT reagent Kit (Takara, Kusatsu, Japan), and amplified using TB Green Premix ExTaq II (Takara) on a Stratagene Mx3000P thermocycler (Agilent Technologies, Santa Clara, CA). The primers for *Cdkn1b* and *Bcl2l11* mRNA were purchased (Takara).

### Database analysis

The genetic alteration of FOXO3, including amplification and deletion, was examined using cBioPortal (https://www.cbioportal.org).

### Mouse experiments

*Gan* mice develop gastric tumors by the activation of Wnt and COX-2/PGE_2_ pathways [26, 27, 42]. Mouse *Foxo3* cDNA was subcloned by RT-PCR using C57BL/6 mouse stomach RNA, mutations at AKT phosphorylation sites (T32A, S252A, and S314A) were introduced using a PrimeSTAR Mutagenesis Basal Kit (Takara), and the ER sequence was ligated to the 3′ end of *Foxo3* cDNA to construct Act-ER FOXO3 cDNA. The construction of *Foxo3*^*Act*^ mice is described in Supplementary Information. To examine the survival ratio, *Foxo3*^*Act/Act*^, *Foxo3*^*+/Act*^*,* and *Foxo3* wild-type mice were treated with Tam (once a week, intraperitoneally [*i.p*.], 2 mg/20 g). *Foxo3*^*Act/Act*^ mice that showed signs of a moribund phenotype and age-matched wild-type mice were euthanized, and blood samples were used for the BUN and CRE tests (Oriental Yeast, Kyoto, Japan). Tissue specimens were histologically examined (*n* = 4 for each genotype). *Gan Foxo3*^*+/Act*^ compound mice were treated with Tam for 40 weeks from 10 weeks of age, and the tumor phenotypes were examined at 50 weeks of age (*n* = 9 for *Gan Foxo3*^+/+^, and *n* = 7 for *Gan Foxo3*^*+/Act*^). Tumor areas were measured on histology sections.

For inhibitor treatment experiments using the PDX model, the same number of gastric cancer-derived organoids (GC19 and GC26) was subcutaneously transplanted into SHO mice. All animal experiments were performed with the protocol approved by the Committee on Animal Experimentation of Kanazawa University.

### Statistical analyses

The data were analyzed using two-sided unpaired *t*-test and are presented as the mean ± standard deviation (s.d.). The data for cell proliferation assay were analyzed by one-way ANOVA test. *p* values of <0.05 were considered to indicate statistical significance.

## Supplementary information

Supplementary Table 1

Supplementary Figures 1-6

Supplementary Materials and Methods
