# Supplementary material for: FOXO3 is a latent tumor suppressor for FOXO3-positive and cytoplasmic-type gastric cancer cells
Source: Oncogene. 2021 Apr 1;40(17):3072–86. doi: 10.1038/s41388-021-01757-x (PMC8084732; doi:10.1038/s41388-021-01757-x)
Supplement: Supplementary file 1 — Supplementary Table 1 [file 41388_2021_1757_MOESM1_ESM.pdf]

Supplementary Table 1. The number of FOXO3 subtypes in each gastric cancer stage

Stage	FOXO3 expression and subcellular localization				Total
	positive			negative (%)	
	FOXO3-Nuc	FOXO3-Cyt	Total (%)		
I	15	11	26 (92.9%)	2 (7.1%)	28
II	2	4	6 (66.7%)	3 (33.3%)	9
III	3	5	8 (72.7%)	3 (27.3%)	11
IV	2	0	2 (100%)	0 (0%)	2
	22	20	42 (84%)	8 (16%)	50
