# Supplementary material for: FOXO3 is a latent tumor suppressor for FOXO3-positive and cytoplasmic-type gastric cancer cells
Source: Oncogene. 2021 Apr 1;40(17):3072–86. doi: 10.1038/s41388-021-01757-x (PMC8084732; doi:10.1038/s41388-021-01757-x)
Supplement: Supplementary file 2 — Supplementary Figures 1-6 [file 41388_2021_1757_MOESM2_ESM.pdf]

# Supplementary Figure 1

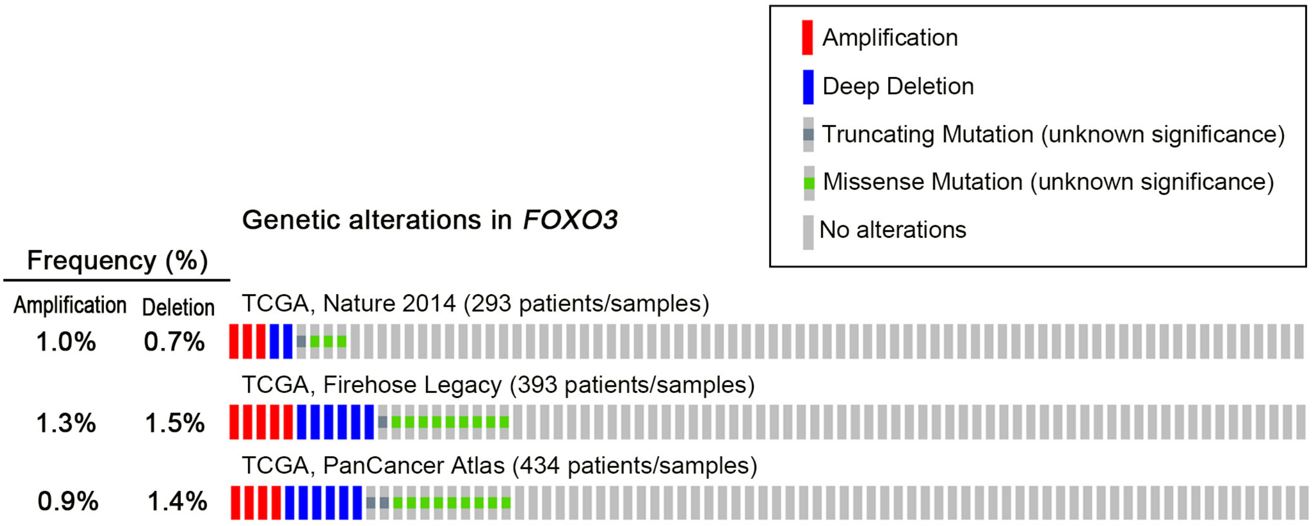


**Supplementary Figure 1.** Database analyses for *FOXO3* genetic alterations in human gastric cancer. The frequencies (%) of amplification and deletion of *FOXO3* gene in each database are indicated.

# Supplementary Figure 2

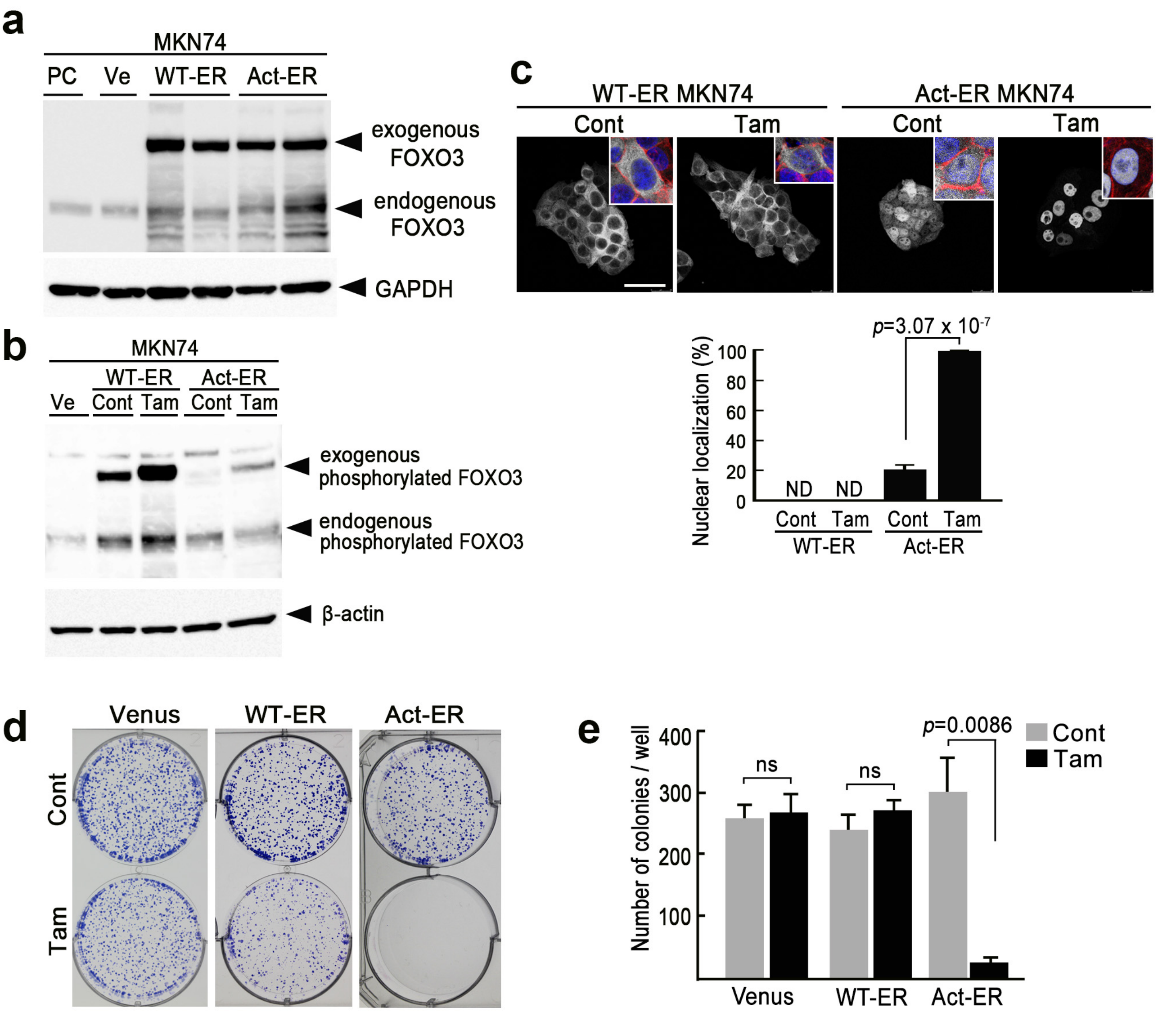


**Supplementary Figure 2.** Growth suppression of MKN74 gastric cancer cells by FOXO3 nuclear accumulation. **a** Immunoblotting results for exogenous FOXO3 (WT-ER and Act-ER) and endogenous FOXO3 in MKN74 cells are shown. PC, Parental cells; Ve, Venus-expressing cells; WT-ER and Act-ER, WT-ER and Act-ER FOXO3-expressing MKN74 cells, respectively. GAPDH was used as an internal control. **b** Immunoblotting results for phosphorylated FOXO3 in control and Tam-treated WT-ER or Act-ER MKN74 cells are shown. β-Actin was used as an internal control. **c** Representative photographs of immunocytochemistry for FOXO3 of the tamoxifen-treated (Tam) and untreated (Cont) WT-ER and Act-ER FOXO3-expressing MKN74 cells (*top*) (Representative images of n=3 independent cultures). Insets show enlarged images with Phalloidin (red) and DAPI (blue) staining. Bar, 50 μm. The ratios of FOXO3-nuclear localized cells in the control and Tam-treated WT-ER and Act-ER FOXO3-expressing MKN74 cells are shown as a bar graph (mean ± s.d.) (*bottom).* A two-sided *t*-test was used to calculate statistical significance, and *p* value is provided. ND, not detected. **d** The results of a colony formation assay of Tam-treated (Tam) and untreated (Cont) MKN74 cells expressing Venus (*left*), WT-ER (*center*) and Act-ER (*right*) in 6-well plates are shown. The images are representative of n=3 independent experiments. **e** The mean colony numbers per well are shown in a bar graph (mean ± s.d.). A two-sided *t*-test was used to calculate statistical difference, and *p* value is provided. ns, not significant.

# Supplementary Figure 3

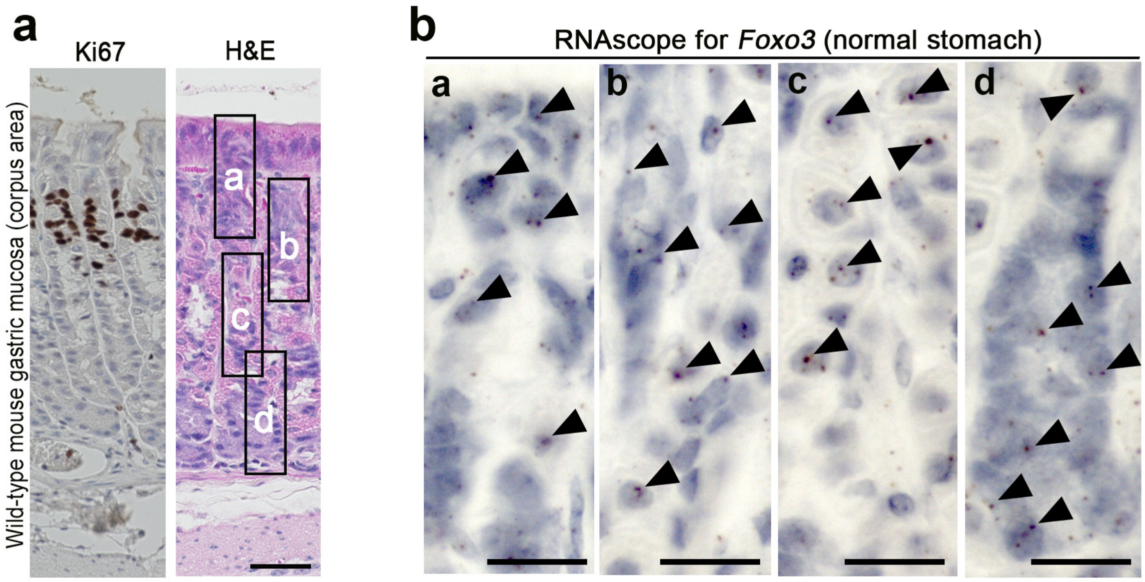


**Supplementary Figure 3.** FOXO3 expression in wild-type mouse stomach. **a** Representative photographs of corpus area of wild-type mouse stomach (H&E) (*right*) and Ki67 immunohistochemistry (*left*). Boxes (a-d) indicate the pit, neck, gland middle, and gland bottom, respectively. Bars, 20 μm. **b** The results of RNAscope *in situ* hybridization for *Foxo3* mRNA in the boxed area in **a** are shown. Arrowheads indicate positive signals. Bars, 20 μm. Images in (**a**) and (**b**) are representative of n=3 biologically independent animals.

# Supplementary Figure 4

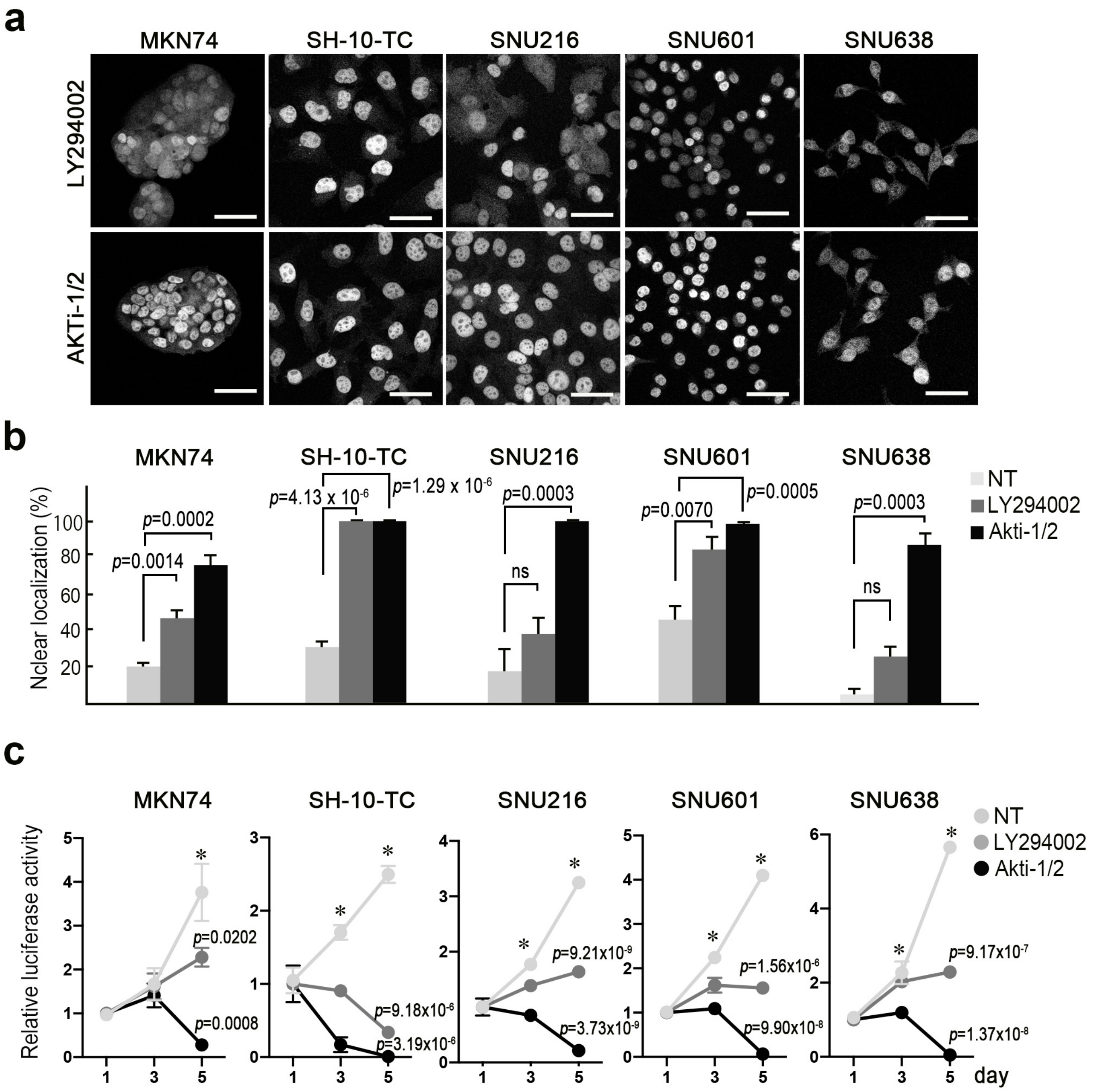


**Supplementary Figure 4.** Growth suppression of gastric cancer cells by PI3K-AKT inhibition. **a** Representative photographs of immunocytochemistry for FOXO3 of the gastric cancer cell lines treated with LY294002 (*top*) and AKTi-1/2 (*bottom*). Bars, 50 μm. The images are representative of n=3 independent experiments. Immunocytochemistry of the untreated control cells are shown in Figure 2b. **b** The ratios of FOXO3 nuclear localization in the indicated gastric cancer cell lines are shown as bar graphs (mean ± s.d.). (n=9 independent microscopic fields for each cell line). NT, no treatment. A two-sided *t*-test was used to calculate statistical significance, and *p* values are provided. **c** Relative cell proliferations examined by luciferase activity in gastric cancer cells with no treatment (NT) and treated with LY294002 or Akti-1/2 are shown (mean ± s.d.). The data at each day point were analyzed by one-way ANOVA test. Asterisks, $p < 0.05$. A two-sided *t*-test was used to calculate statistical difference at day 5, and *p* values vs. NT are provided.

## Supplementary Figure 5

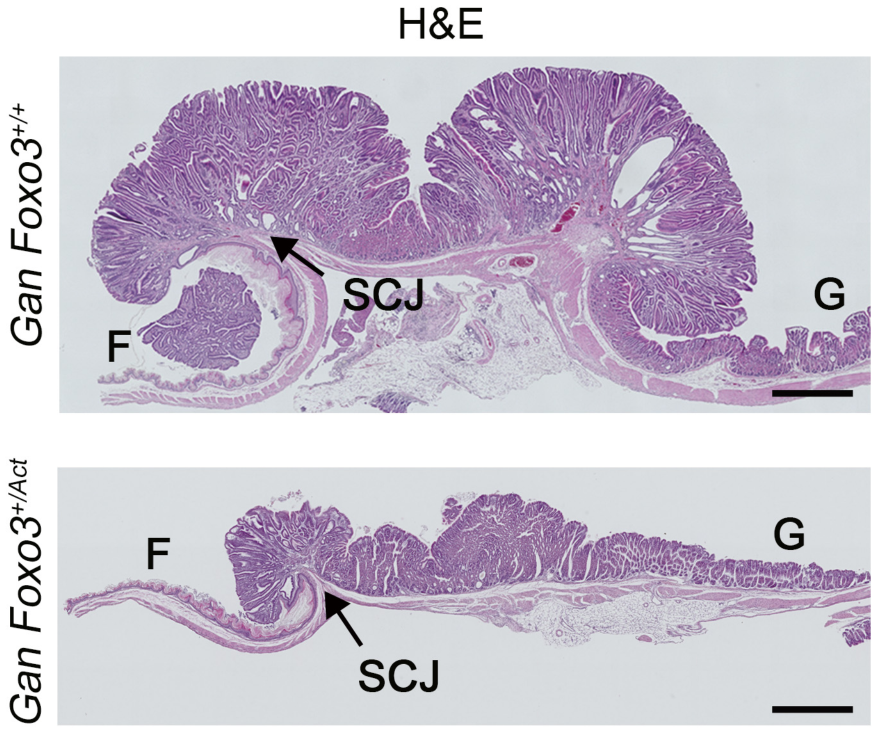


**Supplementary Figure 5.** Representative histology photographs of *Gan Foxo3*$^{+/+}$ (*top*) and *Gan Foxo3*$^{+/Act}$ (*bottom*) mouse gastric tumors (H&E) at 50 weeks of age. F, forestomach; G, glandular stomach; SCJ, squamocolumnar junction. Bars, 1 mm. The images are representative of n=9 and n=7 biologically independent *Gan Foxo3*$^{+/+}$ and *Gan Foxo3*$^{+/Act}$ mice, respectively.

# Supplementary Figure 6

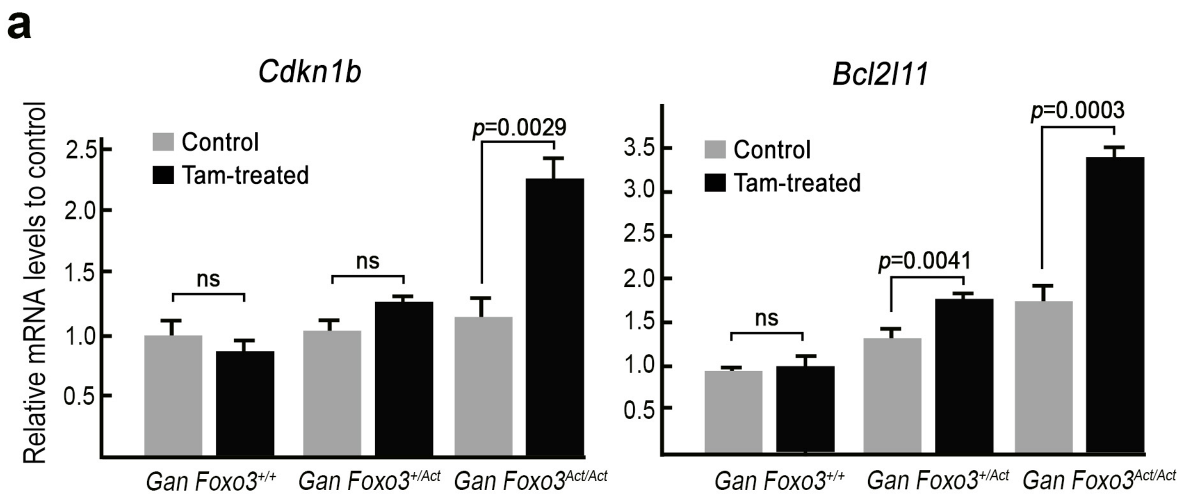


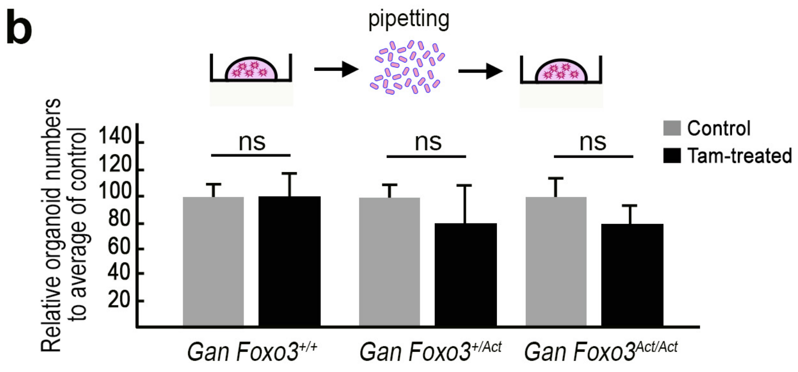


**Supplementary Figure 6.** Induction of FOXO3 target genes by expression of Act-ER FOXO3. **a** The relative mRNA levels of the FOXO3 target genes *Cdkn1b* (*left*) and *Bcl2l11* (*right*) in the control and Tam-treated tumor-derived organoids with indicated genotypes to the mean control level of *Gan FOXO3*$^{+/+}$ organoids (mean ± s.d.). The RT-PCR experiments were repeated five times. **b** A schematic illustration of the mechanical dissociation of organoids by pipetting and passage (*top*). Relative organoid numbers developed after the mechanical dissociation of control and Tam-treated *Gan Foxo3*$^{+/+}$, *Gan Foxo3*$^{+/Act}$, and *Gan Foxo3*$^{Act/Act}$ tumor-derived organoids are shown as a bar graph (mean ± s.d.). The organoid passage experiments were repeated five times. For the data in (**a**) and (**b**), a two-sided *t*-test was used to calculate statistical significance, and *p* values are provided. ns, not significant.
