# Supplementary material for: FOXO3 is a latent tumor suppressor for FOXO3-positive and cytoplasmic-type gastric cancer cells
Source: Oncogene. 2021 Apr 1;40(17):3072–86. doi: 10.1038/s41388-021-01757-x (PMC8084732; doi:10.1038/s41388-021-01757-x)
Supplement: Supplementary file 3 — Supplementary Materials and Methods [file 41388_2021_1757_MOESM3_ESM.pdf]

## Supplementary Materials and Methods

*Antibodies used for immunohistochemistry*

For immunohistochemistry, antibodies against Ki67 (#ab16667, Abcam, Cambridge, UK) and FOXO3 (#12829, Cell Signaling, Danvers, MA) were used as the primary antibody.

*Antibodies used for immunocytochemistry*

For immunocytochemistry, antibodies against FOXO3 (#12829, Cell Signaling) and E-cadherin (#AF748, R&D Systems, Minneapolis, MN) were used as the primary antibody, and Alexa Fluor 488- and Alexa Fluor 594-conjugated antibodies (Thermo Fisher Scientific, Grand Island, NY) were used as the secondary antibody. To detect F-actin in human cancer cells, Alexa Fluor 647-conjugated Phalloidin (Thermo Fisher Scientific) was used.

*Antibodies used for immunoblotting*

Antibodies against FOXO3 (#12829, Cell Signaling, Danvers, MA), phosphorylated FOXO3 at Ser253 (#9466, Cell Signaling), phosphorylated AKT at Ser473 (#4060, Cell Signaling), AKT (#9272, Cell Signaling), β-actin and GAPDH (#281-98721 and #016-25523, FUJIFILM Wako Pure Chemicals), Lamin A/C (#ab133256, Abcam), and α-tubulin (#05-829, Sigma-Aldrich) were used as the primary antibody.

*Construction of WT-ER and Act-ER-expressing cells.*

To construct WT-ER FOXO3- and Act-ER FOXO3-expressing cells, cDNAs from HA-FOXO3a WT-ER and HA-FOXO3a TM-ER plasmid vectors (Addgene #8355 and #8353, respectively) were subcloned to pPB-CAG-IP piggy bac transposon expression vector (kind gift from Hitoshi Niwa, Kumamoto University, Japan) and co-transfected into gastric cancer cells with transposase expression vector. Venus cDNA was subcloned into a pPB-CAG-IP vector as a control.

*Organoid culture medium*

Organoids were cultured in advanced DMEM/F-12 medium with 10 mM HEPES, 2 mM Glutamax, 1×N2, 1×27 (Invitrogen, Carlsbad, CA), N-acetyl-cysteine (Sigma-Aldrich, St. Louis, MO) supplemented with 50 mg/ml EGF (Invitrogen), 100 ng/ml FGF10 (Peprotech, Rocky Hill, NJ), 10 nM gastrin (Sigma-Aldrich), 50% of L-WRN-conditioned medium, 5 μM GSK3 inhibitor (CHIR-99021; Tocris Bioscience, Bristol, UK), and 10 μM ROCK inhibitor (Y-27632; Sigma-Aldrich).

*Construction of Foxo3$^{Act}$ mice*

The targeting vector was designed to express Act-ER FOXO3 under regulation of an endogenous *Foxo3* gene promoter. The targeting vector was electroporated to TT2 mouse embryonic stem cells (kind gift from Yumiko Saga, National Institute of Genetics, Japan), and homologous recombinant clones were injected into blastocysts to generate chimera mice. By crossing chimera mice with C57BL/6 mice, germline transmitted *Foxo3*$^{+/Act}$ mice were obtained.
